# Clinical efficacy of ultrasound-guided stellate ganglion block combined with extracorporeal shock wave therapy on limb spasticity in patients with ischemic stroke

**DOI:** 10.1186/s12883-023-03391-4

**Published:** 2023-10-04

**Authors:** Zhen Yuan, Jun Luo, Qing-feng Cheng, Qiao Zhang

**Affiliations:** 1 Department of Rehabilitation Medicine, The Second Affiliated Hospital of Nanchang University, Nanchang, 330008 Jiangxi China; 2 Rehabilitation medicine and physiotherapy, Medical Department of Nanchang University, Nanchang, 330006 Jiangxi China; 3 Urology Surgery, The Second Affiliated Hospital of Nanchang University, Nanchang, 330008 Jiangxi China; 4 Department of Orthopedics, The Second Affiliated Hospital of Nanchang University, Nanchang, 330008 Jiangxi China

**Keywords:** Stellate ganglion block (SGB), Extracorporeal shock wave therapy (ESWT), Myospasm, Ischemic stroke, Clinical efficacy

## Abstract

**Introduction:**

To observe the clinical efficacy of ultrasound-guided stellate ganglion block (SGB) + extracorporeal shock wave therapy (ESWT) for limb spasticity in patients with ischemic stroke.

**Methods:**

A total of 60 patients with post-stroke limb spasticity in our hospital were selected and randomly divided into four groups (n = 15). In the control group, patients received routine rehabilitation training. Based on routine rehabilitation training, SGB group patients underwent ultrasound-guided SGB, ESWT group patients received ESWT, and SGB + ESWT group patients received ultrasound-guided SGB combined with ESWT. The total treatment course was one month. The Modified Barthel Index (MBI) score, Fugl-Meyer Assessment and upper limb rehabilitation training system were applied to evaluate the activities of daily living, upper limb motor function and upper limb performance before and after treatment. Finally, the improvement after treatment was compared among different groups.

**Results:**

After treatment, compared with the control group, the MBI score and the upper limb score based on Fugl-Meyer Assessment in the SGB, ESWT, and SGB + ESWT groups were significantly increased (*P* < 0.05). Furthermore, compared with the SGB and ESWT groups, SGB + ESWT exhibited a higher upper limb function score (*P* < 0.05), while the MBI score was not significantly different (*P* > 0.05). In terms of upper limb performance ability, patients in the SGB, ESWT and SGB + ESWT groups had better fitting degree, participation and exertion of exercise than those in the control group, and the SGB + ESWT group patients had the same movement trajectory as robots.

**Conclusion:**

Ultrasound-guided SGB and ESWT can reduce the muscle tension of patients, alleviate spasticity, promote the motor function of the upper limb, and improve the working performance of patients. However, the effect of SGB combined with ESWT is better.

## Introduction

Ischemic stroke, an acute cerebrovascular disease, occurs when a vessel supplying blood to the brain is obstructed and causes brain tissue damage [1]. Studies have reported that stroke is the leading cause of death and disability among adults in china [2]. Generally, spasticity is a common complication in patients with stroke, and the clinical symptoms are manifested as the spasticity of hemiplegic upper limb flexor and lower limb extensor [3]. Upper motor neuron damage is a common symptom in patients with stroke, and neuron damage is the key cause for muscle spasticity [4]. In a prospective, longitudinal study of 211 patients with stroke, the incidence of higher spasticity degree in the upper limbs was more frequently than in the muscles of the lower limbs (18.9% vs. 5.5%) [5]. Most of patients with ischemic stroke can return to normal living activities after rehabilitation treatment, but the treatment of post-stroke limb spasticity has always been a difficult problem faced by rehabilitation physicians.

Oral muscle relaxants, botulinum toxin injection, continuous stretching, and low-frequency spasticity therapeutic apparatus are preferred for the treatment of limb spasticity. However, patients receiving the above methods still suffer from limb spasticity repeatedly [6–8]. Stellate ganglion block (SGB) can block peripheral nervous system, inhibit the function of preganglionic and postganglionic fibers at the blocking position, thereby suppressing the muscle tension dominated by the sympathetic nerve fibers in the distribution area [9]. SGB has been widely applied to relieve pain after tumor surgery, alleviate vasospasm and treat heart-related diseases [10–12]. Shock wave is a kind of sound wave with mechanical properties, which can cause a huge change in physical properties such as pressure, temperature and density of the medium [13]. Notably, the shock wave compresses and accumulates the medium rapidly through vibration and high-speed movement, thereby generating energy. In *Chinese guidelines for diagnosis and treatment of acute ischemic stroke 2018*, extracorporeal shock wave therapy (ESWT) serves as a recommended Grade A treatment for post-stroke spasticity [14]. Currently, ESWT has been widely used in clinical practice. In the study of Manganotti et al., half of the 20 patients with stroke treated with shock wave therapy for 12 weeks showed a continuous decrease in muscle tension and didn’t suffer from ESWT-related adverse events [15]. However, there are no clinical reports on SGB + ESWT treating upper limb spasticity in patients with ischemic stroke. Therefore, ultrasound-guided SGB combined with ESWT was employed to treat post-stroke limb spasticity in this study. Additionally, through observing clinical efficacy the combined therapy, this study provided a reference basis for the application of SGB + ESWT in post-stroke limb spasticity patients.

## Materials and methods

### General information

A total of 60 patients diagnosed with ischemic stroke accompanied by limb spasticity in the Second Affiliated Hospital of Nanchang University from January 2022 to December 2022 were selected and randomly divided into the control group (n = 15), SGB group (n = 15), ESWT group (n = 15) and SGB + ESWT group (n = 15). Patients in different groups received different treatments. To be specific, the control group patients underwent routine rehabilitation training; based on routine rehabilitation training, SGB group patients were given ultrasound-guided SGB, ESWT group patients received ESWT, and SGB + ESWT group patients received ultrasound-guided SGB combined with ESWT. The total treatment course of the included patients was one month.

The inclusion and exclusion criteria were shown as follows:

Patients were included if they (1) satisfied diagnostic criteria of *Chinese guidelines for diagnosis and treatment of acute ischemic stroke 2018* [7]; (2) suffered from acute cerebral infarction in light of cranial magnetic resonance imaging (MRI) report; (3) presented their upper limb elbow flexion-extension as grade 2 in Ashworth Scale [16]; (4) had a disease duration of 3–6 months. However, patients were excluded as long as they (1) were accompanied by other severe basic diseases involving the whole body; (2) had mental disorder and failed to cooperate with treatment; (3) may suffer from the recurrence of stroke or other cerebrovascular diseases; (4) were characterized by upper limb fracture or deformity. In this study, informed consent was obtained from the patient or family, and the informed consent form was signed by the guardian of the patients. By the way, this research was approved by the ethics committee of the Second Affiliated Hospital of Nanchang University.

### Therapeutic methods

#### Routine rehabilitation training

The whole included patients who underwent routine rehabilitation training, such as exercise therapy, pneumatic therapy, medium frequency pulsation electrotherapy, and upper limb exercise therapy. The treatment was performed once daily, and the treatment duration was 1 month.

### Stellate ganglion block

A No.7 needle was perpendicular to the skin at the paraesophageal and anterior border of the sternocleidomastoid muscle and about two fingers above the sternoclavicular joint. Under the guidance of the ultrasound, the needle was pushed into the skin. In the sonogram, the needle tip was observed to reach the anterolateral aspect of the transverse process of the seventh cervical vertebra. After the needle was withdrawn a little and no blood was sucked back, 3-4ml of lidocaine was injected. Next, the state of pupil, eyelid, conjunctiva and face of the patient were observed. Besides, the treatment was conducted once every 3 days, and the treatment time was 1 month.

### Extracorporeal shock wave therapy

Firstly, an ESWT device (Suzhou Haobro Medical Device Co., Ltd.: HB101) was selected. Next, the patient was asked to exposure his spastic muscles and fix his body position. Then, a coupling agent was applied to the skin surface of the spastic muscle on the affected side. Subsequently, the spastic muscle groups of patients were positioned, the pressure intensity parameters were set as 1.1–1.3 Bar, the frequency was set as 8–14 Hz, the shock wave probe was moved in the spasm muscle, and the pressure of the handle was about 1-cm skin depression. The shock was performed 3000 times per treatment, and the interval of each treatment was 1 day. Additionally, patients were observed for discomfort before and after treatment, and the treatment duration was 1 month.

### Outcome measure

#### Activities of daily living (ADL) assessment

The Modified Barthel Index (MBI) score [17] was adopted to assess the independence in ADL before and after treatment, including 10 items such as eating, modification, walking (wheelchair), toileting, bed and chair transfer, climbing up and down the stairs, urination control, stool control, dressing and bathing. The score ranged from 0 to 100 points, and a higher score meant better independence in ADL.

### Upper limb function assessment

The Fugl-Meyer Assessment was utilized to appraise the upper limb functions of patients [18]. The score ranged from 0 to 66 points, and a higher score indicated better upper limb functions.

### Performance of upper limb module

The patient was asked to perform an exercise activity using an upper limb robot (Upper Limb Rehabilitation Training System: Armguider-NSS, Shanghai ZD Medical Technology Co., Ltd). The exercise track of the hemiplegic hand was observed. Besides, we also observed the participation, movement strength, and the fitting degree of the exercise track of the robot for patients in the exercise activities.

### Statistical analysis

SPSS 21.0 software was employed for the statistics and analysis of data. Measurement data were expressed as mean ± standard deviation (SD). ANOVA following the Tukey test was used for multi-group comparisons consistent with normal distribution. *P* < 0.05 indicated that the difference was statistically significant.

## Results

### General information about patients

General information of patients was listed in Table 1, including age, disease duration, and body mass index (BMI). There was no significant difference in general information such as onset age, disease duration and BMI between the four groups, indicating that they were comparable (*P* > 0.05).

**Table 1:** Comparison of included patients’ general information

Items	Control group	SGB group	ESWT group	SGB + ESWT group	*P*
Age, years	59.53 ± 7.96	58.73 ± 7.88	60.87 ± 10.84	60.00 ± 9.46	0.93
Disease duration, days	37.27 ± 4.71	36.07 ± 7.38	38.33 ± 7.32	35.93 ± 5.40	0.18
body mass index, Kg/m^2^	23.84 ± 1.65	23.49 ± 1.47	22.79 ± 1.43	23.38 ± 1.48	0.99
MBI scores	42.13 ± 3.44	42.27 ± 3.73	41.73 ± 3.63	41.60 ± 3.91	0.95
Fugl-Meyer motor scale scores	17.07 ± 1.83	16.47 ± 1.60	16.73 ± 1.94	16.40 ± 1.76	0.73

Values are mean ± standard deviation. SGB, stellate ganglion block; ESWT, extracorporeal shock wave therapy; MBI, Modified Barthel Index

### Comparison of MBI score in patients before and after treatment

**Fig. 1:**
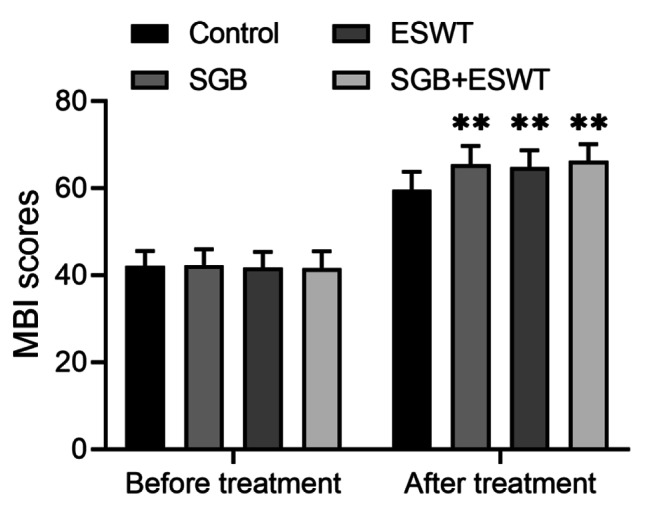
Modified Barthel Index score of patients with stroke before and after treatment MBI, Modified Barthel Index; SGB, stellate ganglion block; ESWT, extracorporeal shock wave therapy; 15 patients per group; **P < 0.01 vs. control group

The MBI score of patients before and after treatment was shown in Fig. 1. Before treatment, the MBI scores between the four groups were not significant (*P* > 0.05). After one month of treatment, compared with the control group, the MBI scores of the SGB, ESWT and SGB + ESWT groups were much higher, and the differences were statistically significant (*P* < 0.05). However, after comparison of the MBI score in the SGB + ESWT group with those in the SGB and ESWT groups, no significant differences were observed among the three groups (*P* > 0.05).

**Comparison of upper limb function scores in patients before and after treatment based on Fugl-Meyer motor scale assessment**.

**Fig. 2:**
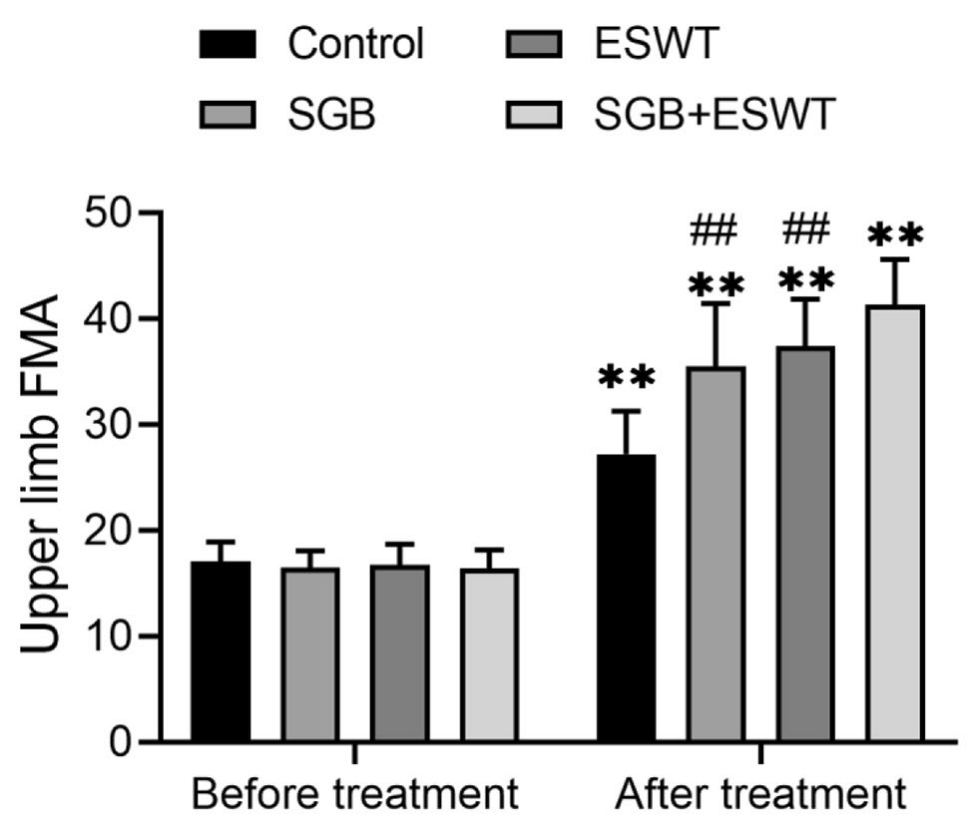
Upper limb function scoring based on Fugl-Meyer Assessment FMA, Fugl-Meyer Assessment; SGB, stellate ganglion block; ESWT, extracorporeal shock wave therapy; 15 subjects per group; **P < 0.01 vs. control group; ^##^*P* < 0.01 vs. SGB + ESWT group

Upper limb function scores of patients before and after treatment based on Fugl-Meyer motor scale assessment were listed in Fig. 2. Prior to treatment, there was no obvious difference in upper limb function scores among the four groups (*P* > 0.05). Upon treatment, compared with the control group, the SGB, ESWT and SGB + ESWT groups showed significantly improved upper limb function scores, and the differences were statistically significant (*P* < 0.05). Additionally, in comparison with the SGB group and the ESWT group, the upper limb function score of the SGB + ESWT group was higher, and the difference was statistically significant (*P* < 0.05).

### Comparison of upper limb performance of patients after treatment

**Fig. 3:**
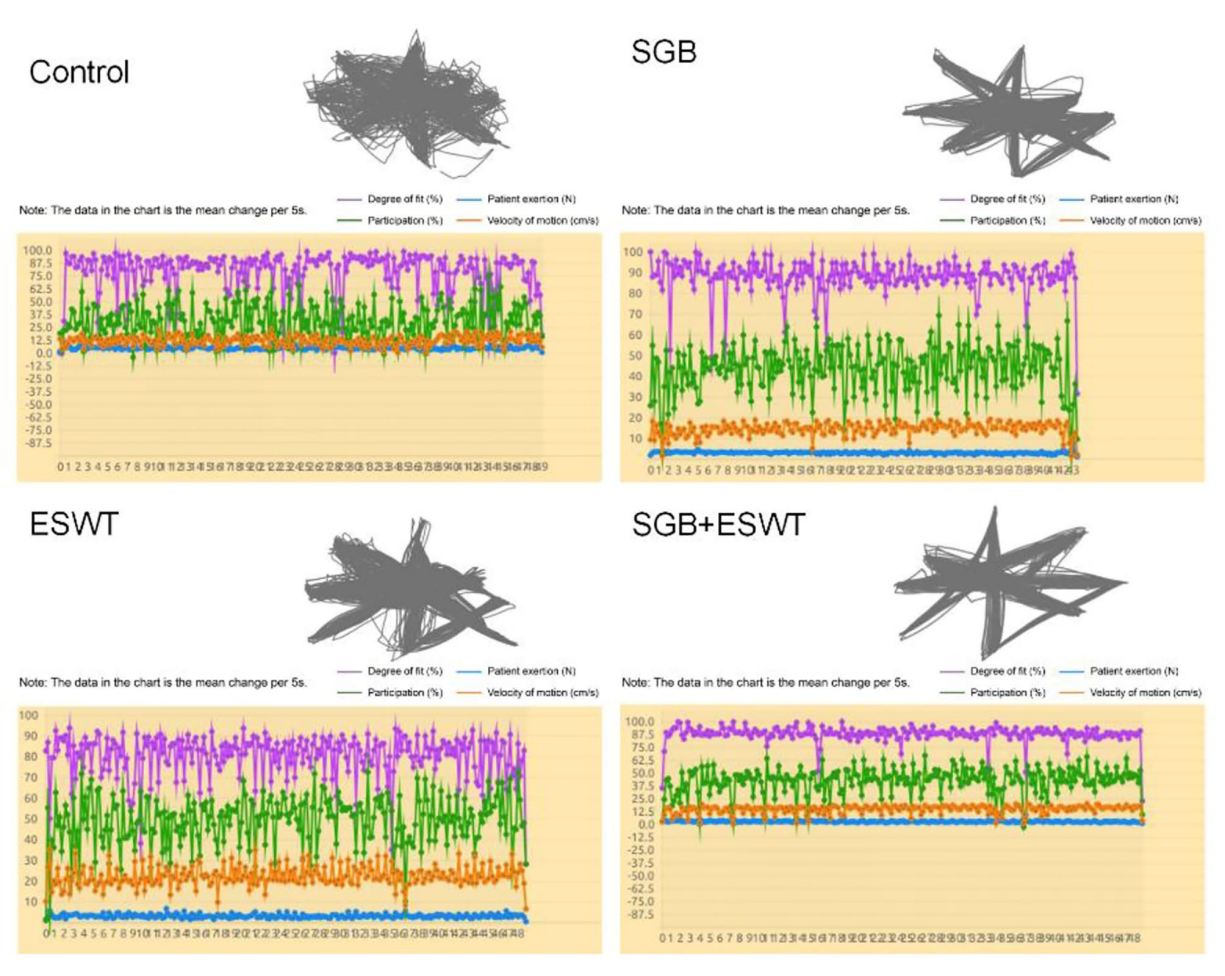
Upper limb performance of patients with stroke The purple curve represented the fitting degree of the patient’ s exercise with the robot, the blue curve indicated the exertion of patients, the green curve represented the participation of patients, and the brown curve indicated the exercise speed of patients. Evaluation criteria: The better the fit, the greater the strength, the better the performance. SGB, stellate ganglion block; ESWT, extracorporeal shock wave therapy

Upper limb performance of patients after treatment was exhibited in Fig. 3. After comparing the exercise track of the robot and patient, we observed that the fitting degree of exercise of patients in the SGB, ESWT and SGB + ESWT groups was significantly better than that in the control group. Furthermore, the exercise track of the patients in the SGB + ESWT group was basically the same as that of the robot. Moreover, SGB + ESWT group patients showed better participation and exertion of exercise than SGB group and ESWT group patients.

## Discussion

Ischemic stroke is characterized by high mortality, disability, and recurrence rate. The bulk of patients with stroke are accompanied by limb spasticity, seriously affecting their limb motor functions [19]. For one thing, clinical drugs and physical methods for treating spasticity are not ideal due to defects like short curative effect, easy recurrence, and complex operation. For another, oral muscle relaxants are not conducive to rehabilitation training because they bring muscle relaxation and weakness to patients [6]. Though botulinum toxin injection can quickly alleviate the symptoms of limb spasticity in patients, the long-term effect is not satisfactory [7]. Therefore, finding an effective, durable and convenient anti-spasticity therapy is of great significance for the rehabilitation of patients.

In this research, both SGB and ESWT treatments could reduce the muscle tension and improve the upper limb motor functions of patients. After treatment, the SGB, ESWT and SGB + ESWT groups not only exhibited obviously higher MBI score but also presented a significantly increased upper limb function score based on Fugl-Meyer motor scale assessment, relative to the control group. Besides, compared with the SGB and ESWT groups, the upper limb function score was a bit higher in the SGB + ESWT group, while the MBI score was not significantly different. As for performance of upper limb exercise, patients in the SGB, ESWT and SGB + ESWT groups had significantly better fitting degree, participation and exertion than those in the control group. Moreover, the exercise track of the patients in the SGB + ESWT group was the same as that of the robot basically. Shortly speaking, ultrasound-guided SGB combined with ESWT has achieved good results in the treatment of limb spasticity in patients with ischemic stroke.

SGB plays a role in regulating the functions of the vegetative nervous system, endocrine system and immune system [12, 20–22]. For example, SGB contributes to maintaining the stability of the internal environment in the body, thereby allowing the treatment of many vegetative nervous system disorders. Currently, most scholars believe that the block effect of stellate ganglion is mainly manifested in two aspects: central nervous system and peripheral nervous system. On the one hand, SGB regulates the stability of the thalamic environment so that the vegetative nervous function, endocrine function and immune function of the body remain normal [23]. On the other hand, the effect of SGB on the peripheral nervous system is achieved because the function of the preganglionic and postganglionic fibers at the blocked site is inhibited, as well as cardiovascular movement, glandular secretion, muscular tone, bronchoconstriction, and pain transmission innervated by the sympathetic nerve fibers at the site of distribution. Such an effect of SGB on the peripheral nervous system allows it to treat some conditions related to the head and neck, upper limbs, shoulders, heart and lungs [24–26]. In an animal experiment, SGB could effectively improve the cerebral ischemia and neurological function of diabetic rats with ischemic stroke, and the main mechanism was that SGB reduced the phosphorylation of NF-κB p65 and inhibited inflammatory response [27]. According to the above findings, SGB may help improve brain blood circulation, nourish damaged brain neurons, improve the blood supply of the limbs, relieve muscle spasticity, promote tissue metabolism and restore the limb functions for the patients suffering stroke by regulating the function of central and peripheral nerves [28]. As for the shock wave, it utilizes the principles of hydro-electric energy conversion and transmission to generate energy gradient difference and torsional tension between tissues with different densities, thereby causing a series of biochemical effects in tissues and cells [29]. ESWT has been reported to possess the following biological effects: tissue damage repair and reconstruction, tissue adhesion release, vasodilation and vascular regeneration, analgesia and nerve termination closure, high-density tissue lysis, inflammation and infection control, etc. [30]. Moreover, study proved that extracorporeal shock wave has a certain effect on relieving spasticity. MARIOTTO et al. believed that extracorporeal shock waves exerted functions by inducing the synthesis of nitric oxide (NO) [31]. Actually, extracorporeal shock waves induce the synthesis of NO by vascular endothelial cells. Also, NO is involved in the formation of neuromuscular junctions. Extracorporeal shock waves increase the formation of neuromuscular junctions, contributing to regulating neurotransmitter and synaptic plasticity, thereby promoting the physiological function of the central nervous system [32, 33]. Moreover, shock wave pressure can reduce spasticity by reducing the stiffness and fibrosis of spastic muscle connective tissue through breaking the functional relationship between actin and myosin [34]. In addition, the study by Kenmoku et al. claimed that ESWT could reduce the spasticity of wrist and elbow flexor, which was consistent with the results of this study [35].

There are some shortcomings in this paper. Firstly, the sample size included in this study was small, so a multi-center study with a large sample size will be conducted in the future. Secondly, this study only preliminarily explored the clinical efficacy of SGB combined with ESWT in the treatment of limb spasticity in patients with stroke rather than elucidated the specific mechanism, so further research is needed. Last but not least, for various reasons, neither the statistics for the adverse reactions and long-term effects nor the further evaluation for the safety of SGB combined with ESWT for the treatment of limb spasticity in patients with stroke was carried out. Despite the above demerits, this study still contributes to the clinical application of SGB + ESWT in treating limb spasticity in patients with stroke and provides a relevant reference basis for subsequent studies.

## Conclusions

Ultrasound-guided SGB combined with ESWT can effectively alleviate the increase in post-stroke limb muscle tension, alleviate upper limb spasticity, ameliorate upper limb motor function, improve the performance ability and restore the limb function of patients. Moreover, ultrasound-guided SGB + ESWT provides new ideas for clinicians and rehabilitation therapists to effectively reduce the limb spasticity of patients. All in all, SGB + ESWT can benefit more patients with stroke with limb spasticity and is worthy of clinical application.

## Data Availability

The data that support the findings of this study are available on request from the corresponding author.

## Abbreviations

SGB: stellate ganglion block
ESWT: extracorporeal shock wave therapy
SD: standard deviation
BMI: body mass index
MBI: Modified Barthel Index
ADL: Activities of daily living
